# Progress of Dental Surgery

**Published:** 1851-01

**Authors:** 


### EDITORIAL DEPARTMENT.

*Progress of Dental Surgery.*—Since the commencement of the present century, Dental Surgery has progressed with most astonishing rapidity. Previously to this time, although much had been written upon the subject in France, and a little in England, its resources had scarcely begun to be developed. It was looked upon as a mere mechanical art, requiring but little preparatory instruction, and so few were engaged in its practice, that scarcely one person in a hundred had ever heard of a dentist. The operation of filling a tooth was rarely performed, the duties of the dentist consisting, for the most part, in the extraction of an aching tooth, the removal of tartar, and occasionally in replacing the loss of the natural teeth, with artificial substitutes, rudely carved from the ivory of the hippopotamus or elephant.

There were not in 1800, more than fifteen or twenty dentists in the United States, and nearly all of these were uneducated men, totally ignorant of the anatomy of the human body, and of the laws of health and disease, their whole stock of professional knowledge consisting in what they had learned from a few awkwardly performed operations, which in a large majority of the cases, had been productive of more injury than good to those upon whose teeth they had been performed. Gardette of Philadelphia, was almost the only medically educated dentist at that time in the western world, and almost the only one capable of treating the diseases of the mouth in a judicious and skillful manner.

Now, in nearly every city, town and village in the United States, the services of the dentist are almost as frequently sought as those of the physician; and instead of fifteen or twenty, we have twenty-five or twenty-six hundred practitioners of dental surgery. But it must at the same time be confessed, that a large majority of those who call themselves dentists have not a single qualification entitling them to the appellation. They have assumed the claim, in too many instances, with but little or no preparatory instruction or study, depending for practice rather upon bold and unblushing impudence in setting forth pretensions to ability, which even the best informed and most skillful do not possess, than real merit. Still there are ***very many*** well educated and scientific men in the profession—men who would be an honor and ornament to any calling; and it is to the labors and influence of such individuals that it is indebted for the dignified position it now occupies in public estimation.

With the exception of the works on the diseases of the mouth, the late Dr. Gardette probably brought with him from France, the only treatises on the teeth accessible to the profession in this country fifty years ago, were those of the celebrated John Hunter and Berdmore, and it is doubtful whether more than a single copy of either of these had found its way across the Atlantic. Then we had no literature of our own, and no facilities for the acquisition of a knowledge of the art as it existed at *that* time, and instruction could only be had at the expense of more time, labor and money than any one would have been justified in incurring. Consequently, experience was the only available teacher that could be had, and it would be difficult to estimate the amount of injury and suffering inflicted upon those who had the misfortune to fall into the hands of the self-taught practitioners of that day. Had they been as numerous as are the ignorant pretenders of the present, it would, if the field for their manipulation had been as ample, have been incalculable.

Now we have text books of our own, and translations and re-publications of nearly every European work upon the diseases of the mouth, of any value. Besides, we have four periodicals devoted to the interests of the profession, two colleges in successful operation, and a third has recently been chartered. The rapidity with which one improvement or discovery has followed another in the construction and application of dental substitutes, as well as in every other description of appliance employed in this branch of surgery, during the last twenty years, has at least placed *practical dentistry* side by side with the other branches of manual medicine.

But, notwithstanding the high state of excellence to which operative and mechanical dentistry have arrived, the art is not practiced by a large number even of the better class of practitioners, as successfully as it might be. It is too generally regarded as a mere mechanical pursuit, and as such, rather than as a science and art, it is chiefly cultivated and practiced. The Greek motto, "Not the theory but the practice," is too universally adopted by dentists. The latter, to be successfully applied, should be based upon the former. Although theory, so far as the treatment of disease is concerned, is deduced from, and its truth proven, by practice, it constitutes the only safe guide to it. To be able to fill a tooth in the neatest and most substantial manner, or to construct a dental substitute in a style of unsurpassed beauty, is not the only qualification necessary for the dentist. There are circumstances under which an operation upon the mouth cannot be *beneficially* or even *safely* performed. He should be capable, therefore, of discriminating correctly between the indications and counter-indications of treatment. He should know *when,* as well as *how,* an operation should be performed. Without such knowledge he will be liable constantly to commit the most egregious blunders, and in the long run, to do more injury than good.

We would not have it understood by what we have said, that we consider surgical skill and mechanical ability of *minor importance,* as they are both *indispensable* to a *good practitioner;* but what we mean is this, that they would be exercised more efficiently if guided by a thorough knowledge of the science of health and disease. Entertaining this opinion, we cannot but believe that if dentists were as ambitious to excel in the medical sciences generally, as many, very commendably, are, in mechanical manipulations, the profession would have stronger claims to public confidence, and occupy a far more enviable and elevated position than it now does. A dentist cannot be too thoroughly skilled in the surgical and mechanical part of his profession, and every day furnishes abundant proof that most practitioners are sadly deficient in these departments, but as a general thing they are still more deficient in medical knowledge. This should not be, and we trust the time is not distant when most dentists will be as thoroughly accomplished in anatomy, physiology, pathology and therapeutics, as the practitioners of any other specialty of medicine.

